# Is Health-Related Quality of Life Associated with Upper and Lower Airway Inflammation in Asthmatics?

**DOI:** 10.1155/2013/539290

**Published:** 2013-08-31

**Authors:** Nicola Scichilone, Fulvio Braido, Salvatore Taormina, Elena Pozzecco, Alessandra Paternò, Ilaria Baiardini, Vincenzo Casolaro, Giorgio Walter Canonica, Vincenzo Bellia

**Affiliations:** ^1^Dipartimento Biomedico di Medicina Interna e Specialistica (DIBIMIS), Sezione di Pneumologia, University of Palermo, 90146 Palermo, Italy; ^2^Allergy & Respiratory Diseases Clinic, DIMI, University of Genoa, IRCCS AOU San Martino-IST, 16132 Genova, Italy; ^3^Dipartimento di Medicina e Chirurgia, University of Salerno, 84081 Salerno, Italy

## Abstract

*Background.* Allergic diseases impair health-related quality of life (HR-QoL). However, the relationship between airway inflammation and HR-QoL in patients with asthma and rhinitis has not been fully investigated. We explored whether the inflammation of upper and lower airways is associated with HR-QoL. *Methods.* Twenty-two mild allergic asthmatics with concomitant rhinitis (10 males, 38 ± 17 years) were recruited. The Rhinasthma was used to identify HR-QoL, and the Asthma Control Test (ACT) was used to assess asthma control. Subjects underwent lung function and exhaled nitric oxide (eNO) test, collection of exhaled breath condensate (EBC), and nasal wash. *Results.* The Rhinasthma Global Summary score (GS) was 25 ± 11. No relationships were found between GS and markers of nasal allergic inflammation (% eosinophils: *r* = 0.34, *P* = 0.24; ECP: *r* = 0.06, *P* = 0.87) or bronchial inflammation (pH of the EBC: *r* = 0.12, *P* = 0.44; bronchial NO: *r* = 0.27, *P* = 0.22; alveolar NO: *r* = 0.38, *P* = 0.10). The mean ACT score was 18. When subjects were divided into controlled (ACT ≥ 20) and uncontrolled (ACT < 20), the alveolar NO significantly correlated with GS in uncontrolled asthmatics (*r* = 0.60, *P* = 0.04). *Conclusions.* Upper and lower airways inflammation appears unrelated to HR-QoL associated with respiratory symptoms. These preliminary findings suggest that, in uncontrolled asthma, peripheral airway inflammation could be responsible for impaired HR-QoL.

## 1. Background

Asthma is one of the most common chronic diseases in the world and is associated with high morbidity, decreased productivity, poorer quality of life, and higher health care costs. According to the Global Initiative for Asthma (GINA) guidelines [1], the goal of asthma therapy is to achieve and maintain full control over the disease. Clinical manifestations of asthma can be controlled with appropriate anti-inflammatory treatment. When asthma is controlled, there should be no more than occasional flare-ups. Moreover, it would be desirable to accomplish full asthma control using the minimum possible number of drugs to reduce the likelihood of side effects. In other words, the goal of GINA guidelines is the return to an “acceptable” health related (HR) quality of life (QoL), with little or no impact of the disease on patient's life.

An important factor that may impact on current strategies of asthma control is the presence of associated morbidities. Among these, rhinitis plays a prominent role [2]. The association of rhinitis and asthmatic symptoms reduces performance at school and work, affects learning, and worsens the quantity and quality of sleep. For all these reasons, in recent years a growing number of studies have focused on assessing the HR-QoL benefits that medical care can provide to patients with asthma and associated conditions [3, 4]. Several studies have demonstrated a correlation between the severity of rhinitic and asthmatic symptoms, as well as the impact of associated rhinitis on asthma severity [2, 5–7]. In addition, allergic rhinitis and asthma have been demonstrated to interfere with HR-QoL [4, 7, 8]. However, to what extent the severity of upper and lower inflammation affects HR-QoL in patients with asthma and rhinitis has not been investigated so far. The current study aimed at exploring whether HR-QoL of patients with respiratory allergy diseases is associated with inflammatory changes in the upper and lower airways. Therefore, it is intended as preliminary to larger, more specifically tailored studies. 

## 2. Methods

### 2.1. Patients

The study was conducted at the Department of Biomedicine and Internal Medicine, Section of Pneumology, University of Palermo upon approval from the local research ethics committee. We recruited subjects with bronchial asthma and concomitant allergic rhinitis, on the basis of clinical assessment and skin reactivity to common allergens. The diagnosis of asthma was based on the presence of respiratory symptoms such as cough, chest tightness, wheezing, acute episodes of dyspnea, and the ascertainment of variable airway obstruction by chest examination and spirometry, in agreement with the GINA guidelines [1]. Allergic rhinitis was diagnosed by the presence of recurrent episodes of runny nose, sneezing, nasal congestion, and itching, according to the most recent ARIA guidelines [9]. Skin prick tests were performed on all subjects at least one week after suspension of antihistamine therapy. Subjects enrolled in this study had mild asthma (FEV_1_ > 80% predicted) and tested positive to at least one aeroallergen. We excluded patients with heart disease and/or uncontrolled chronic liver disease and cancer, both past and current. 

### 2.2. Study Design

The study was performed in one visit. Each patient underwent a clinical evaluation and the administration of questionnaires on HR-QoL assessment (Rhinasthma) and the degree of disease control (Asthma Control Test, ACT). Subjects were refrained from taking medications the two weeks preceding the study day, with the exception of rescue medications. Each patient also underwent the following functional and biological investigations: respiratory function test, measurement of exhaled nitric oxide (eNO), collection of exhaled breath condensate (EBC), and nasal wash. Short-acting beta-agonists were stopped the day before the tests. The study was performed in accordance with the Good Clinical Practice guidelines recommended by the International Conference on Harmonization of Technical Requirements and was approved by the Ethics Committee of University Hospital of Palermo, and all participants gave written informed consent before inclusion. All measurements took place in the morning, and coffee or tea was not allowed prior to the evaluation. 

#### 2.2.1. Clinical Investigations

The Rhinasthma questionnaire assesses the impact of rhinitis and asthma on the patient's HR-QoL. This questionnaire is composed of 30 questions, whose responses are graded based on the subject's perception of symptoms and converted into a 0–100 score to allow a quantitative estimate of the QoL associated with disease of the upper airways (UA) or lower airways (LA) and the impact of allergic symptoms over the QoL (Rhinitis-Asthma Impact (RAI)). Moreover, Rhinasthma provides a global summary (GS) that indicates the overall impact of symptoms over the patient's QoL [8].

Each patient was also administered the ACT questionnaire which allows the estimate of the level of asthma control. The test consists of five questions. The resulting ACT score is interpreted as follows: fully controlled asthma (score ≥ 25), poorly to partially controlled asthma (score 20–24), or uncontrolled asthma (score < 20) [10]. The questions address symptoms and rescue medication use occurring within the 4 weeks preceding the evaluation.

#### 2.2.2. Functional Investigations

Respiratory function was assessed through a bell-shaped spirometer, in line with the most recent guidelines of the ATS/ERS recommendations [11]. We recorded, in addition to FEV_1_, FVC, and FEV_1_/FVC, also the expiratory flows at different lung volumes (FEF_25%_, FEF_50%_, FEF_75%_, and FEF_25−75%_). Lung function was defined as normal when FEV_1_/FVC was above 88% of predicted (lower limit of normality).

#### 2.2.3. Biological Investigations

This evaluation aimed at assessing the inflammatory profile of lower and upper airways by employing nitric oxide (NO) measurements in the expired air, exhaled breath condensate (EBC), and nasal wash. 

The fraction of exhaled NO (FENO) was measured with a standardized single-breath method by using the electrochemical analyzer (HypAir FeNO, Medisoft), which was calibrated daily. The measurement procedure was consistent with guidelines published by the American Thoracic Society/European Respiratory Society [12]. Subjects were in sitting position during the entire procedure. Each individual was asked to take a deep inspiration from room air and, immediately after, to forcefully exhale through the mouthpiece that was connected to the online analyzer. To determine if subjects maintained a constant flow-rate during exhalation, a continuous monitoring of expiratory flow rate by graphic display on the monitor screen was used. All samples were acquired in the morning between 11:00 and 12:00. FENO was obtained as the mean of three acceptable measurements at the airflow rate of 50 mL/s. The alveolar concentration of NO (Calv) was calculated by using multiple exhalation flow rates of 50, 150, 250, and 350 mL/s. Calv is described by the slope of the plotting of the expiration rates of NO against the exhalation airflow. No correction formula was applied, since this is still a matter of debate. 

EBC was collected using a condenser (EcoScreen; Jaeger; Wurzburg, Germany) [13]. Patients were asked to clean the oral cavity before and during operation to reduce contamination of the sample with saliva. The subjects were asked to breathe at tidal volume, with a normal frequency, through a mouthpiece connected to a two-way valve with saliva trap, wearing a nose clip for about 10 minutes. The condensate, whose quantity varied between 1 and about 4 mL, was collected on ice at −20°C, aliquoted, and transferred immediately at −80°C. The samples were analyzed within one month of collection. The pH of these samples was measured with a pH meter (*HI 221, HANNA Instruments*) equipped with a microelectrode after deaeration with argon (350 mL/min) for 5 minutes to remove carbon dioxide. 

Nasal washes were performed by instilling 5 mL of Ringer solution, preheated at 37°C, in each nostril through a sterile syringe. During the procedure the patient sits with head tilted back at 30 degrees horizontally and occludes the nasopharynx with the soft palate, then tilts his head forward and forces the liquid into a sterile test tube. The volume of liquid obtained was measured using a graduated container, then the sample was stirred and filtered through a Cell Strainer (70 *µ*m mesh, BD Falcon) to remove mucus and centrifuged at 1600 rpm at 4°C for 20 minutes. The supernatant was stored at −80°C for subsequent determination of eosinophil cationic protein (ECP), while the cellular pellet was resuspended in 0.3 mL of PBS. An aliquot (20 *μ*L) of this suspension was applied onto a Burker chamber for the total cellular count, then equivalent numbers of cells were cytospun onto slides for differential cell count (Shandon) at 500 rpm for 5 minutes, allowed to dry, and stained with Diff-Quick. Slides were observed at the optical microscope by two different operators in order to verify the reproducibility of the data. At least 100 cells were enumerated in 3 different sites or 6 random fields. ECP concentration was measured in supernatants by chemiluminescence using an IMMULITE 2000 instrument as described. ECP concentration is expressed as ng/mL. The limit of the assay is 200 ng/mL.

#### 2.2.4. Statistical Analyses

Due to the explorative nature of the study, no formal sample size calculation was performed. Depending on the distribution of the variables, nonparametric tests were used whenever appropriate. Data are reported as mean ± SD. Nonparametric tests were used for nonnormally distributed variables. Unpaired *t*-test was used to compare variables from different groups. Linear regression slopes or Spearman rank correlations were constructed to assess the relationships between variables. In all analyses, two-tailed *P* values ≤ 0.05 were considered statistically significant. The statistical package we employed was StatView 5.0.1 (Abacus Concept, Berkeley, CA, USA). 

## 3. Results

**Figure 1:**
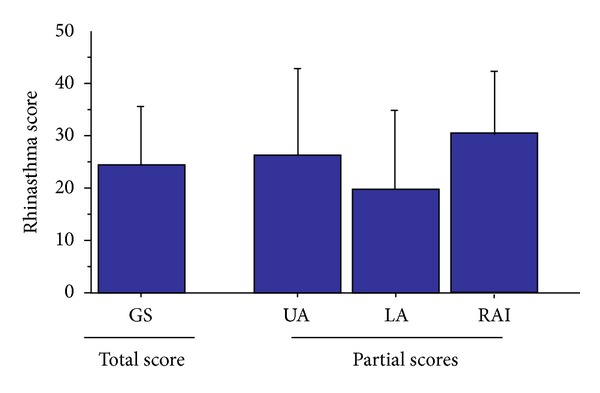
Values for (single) domains of the Rhinasthma questionnaire. GS: Global Summary score; UA: upper airway score; LA: lower airway score; RAI: rhinitis-asthma impact score.

Twenty-five consecutive asthmatics were recruited. After enrollment, three subjects refused to participate for reasons not related to the nature of the study. A total of twenty-two subjects took part in the study (M/F: 10/12, age: 38 ± 17 yrs, mean ± SD). The GS score of Rhinasthma was 25 ± 11, ranging between 7.5 and 47.5.  Figure 1 also depicts the mean ± SD values of single domains (UA, LA, and RAI). All patients showed values of respiratory function within a normal range (FEV_1_/FVC% predicted above 88%, LLN). FEV_1_% predicted ranged from 82 to 129%, and FVC% predicted ranged from 83 to 129%. Complete baseline lung function measures of the study subjects are presented in Table 1. 

The biological findings from upper and lower airway procedures are described in Table 2. With regard to the NO values, the FENO values were around the upper normal level of eNO, which ranges from 20 to 30 ppb, with 95th percentile values being 39 ppb for subjects 12 to 80 years of age. On the other hand, no reference values are still available for the alveolar concentrations. The pH of the EBC was lower than that usually recorded in healthy subjects, suggesting a condition of airway inflammation. The detection of ECP levels and eosinophils in the nasal wash also confirm the occurrence of allergic inflammation in the upper airways. We also assessed the level of asthma control: the mean ACT score was 18; in detail, 3 patients had a totally controlled asthma (ACT = 25), 6 partially controlled (20 ≤ ACT < 24), and 13 uncontrolled (ACT < 20).

**Figure 2:**
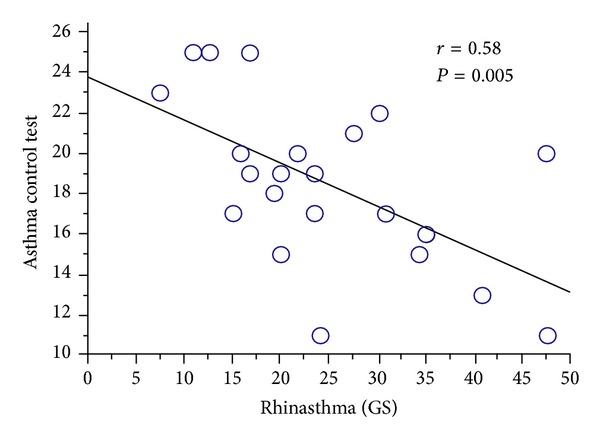
Correlation between the Rhinasthma total score and the Asthma Control Test score.

**Figure 3:**
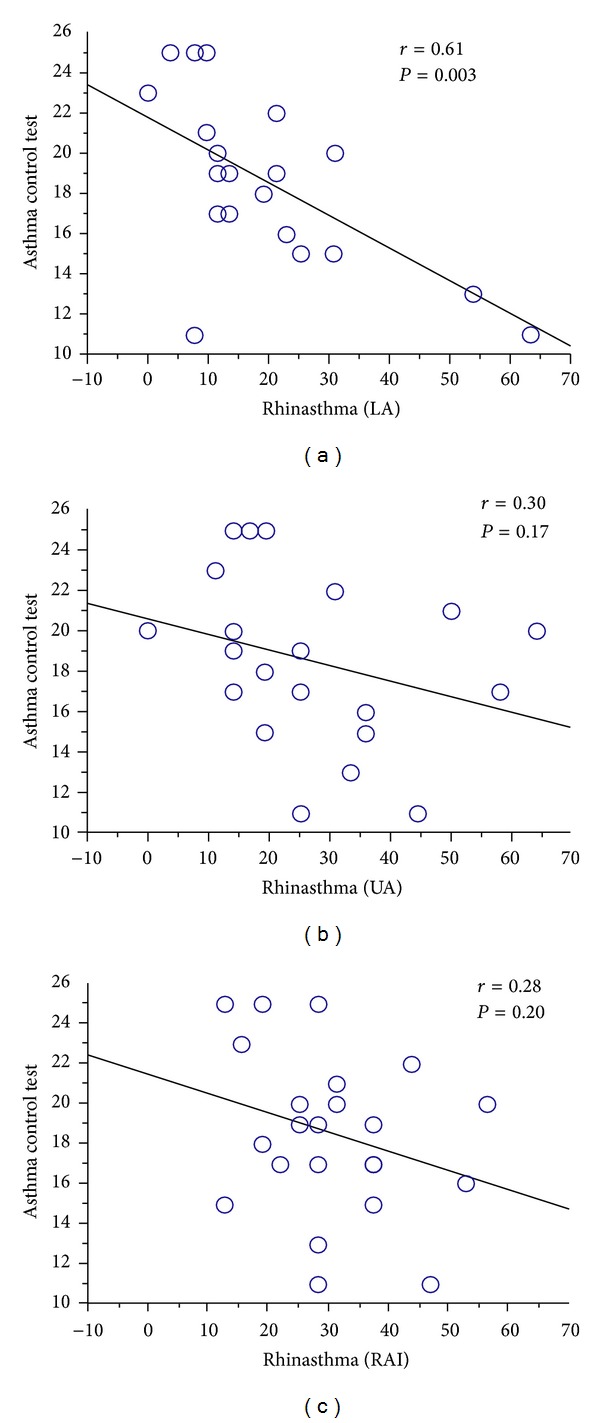
Correlations between the Asthma Control Test and Rhinasthma LA (a), UA (b), and RAI (c). LA: lower airway score; UA: upper airway score; RAI: rhinitis-asthma impact score.

As expected, we found a significant correlation between the Rhinasthma GS score and the ACT score (*r* ^2^ = 0.34, *P* = 0.005) (Figure 2). In addition, Rhinasthma LA (*r* ^2^ = 0.37, *P* = 0.003) (Figure 3(a)) but not UA (*r* ^2^ = 0.09, *P* = 0.17) and RAI (*r* ^2^ = 0.08, *P* = 0.20) (Figures 3(b) and 3(c)) correlated significantly with the ACT score.

To assess whether the HR-QoL is affected by the degree of upper and lower airway inflammation, we explored possible correlations between these measures through simple regression analysis. The Rhinasthma GS did not correlate with the percent of eosinophils (*r* ^2^ = 0.11, *P* = 0.24), or with ECP levels (*r* ^2^ = 0.004, *P* = 0.87) in the nasal wash. Likewise, we found no significant correlation between Rhinasthma GS and the EBC pH (*r* ^2^ = 0.01, *P* = 0.44). Finally, neither FeNO or Calv values showed a significant correlation with the Rhinasthma GS. Similar results were obtained for the single domains of Rhinasthma (UA, LA, and RAI). 

**Figure 4:**
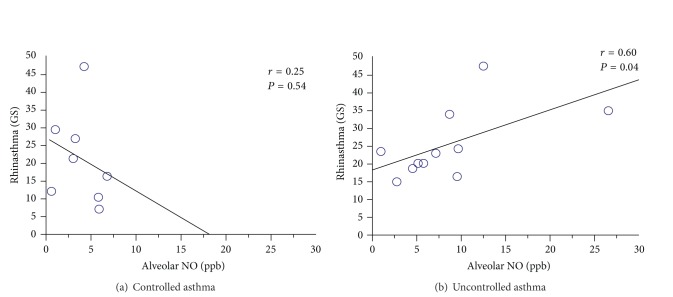
Correlations between the Rhinasthma global score and alveolar NO in controlled (a) and uncontrolled (b) asthmatics.

To further investigate the role of airway inflammation on the HR-QoL of patients with asthma, we divided them into two groups according to the level of disease control: group 1 (patients with fully or partially controlled asthma, ACT ≥ 20) and group 2 (patients with uncontrolled asthma, ACT < 20). The two groups were similar with respect to the pulmonary function: FEV_1_ was, respectively, 105 ± 18% and 95 ± 22% of predicted value, *P* = 0.26; FVC was 116 ± 20% and 101 ± 17%, *P* = 0.07. Similarly, the expiratory flow rates at various lung volumes did not differ significantly between the two groups (*P* > 0.05 in all cases). With regard to the inflammatory markers in the nasal washes, no significant differences were found between groups 1 and 2 in eosinophil percentages (15 ± 9.8% versus 13 ± 8%, respectively; *P* = 0.67) and ECP concentrations (5.8 ± 3.2 ng/mL versus 3.9 ± 3.6 ng/mL, respectively; *P* = 0.42). The pH of EBC was, respectively, 6.85 ± 0.16 and 6.79 ± 0.20 (*P* = 0.49). FeNO levels were not significantly different between the two groups (16 ± 5.3 ppb versus 25 ± 23 ppb, respectively; *P* = 0.26). In contrast, Calv was threefold higher in patients with uncontrolled asthma (9 ± 6.5 ppb) than in those with totally or partially controlled asthma (3 ± 2.3 ppb) (*P* = 0.01). In addition, a significant correlation between Rhinasthma GS and Calv was found in patients with uncontrolled asthma (*r* = 0.60, *P* = 0.04) (Figure 4).

## 4. Discussion 

The results of this pilot study suggest that, in individuals with uncontrolled asthma, the HR-QoL is associated with inflammatory changes in the peripheral airways. The findings of the current study could carry clinical implications. In our previous study [14], we demonstrated that the improved control of respiratory symptoms obtained with nasal corticosteroids in allergic rhinitics with asthma appeared to be mediated by functional changes in the peripheral airways. It is plausible to speculate that, in individuals with uncontrolled asthma, a more intensive anti-inflammatory treatment, perhaps targeting the small airways, is necessary to improve HR-QoL. 

The main limitation of the study is represented by the small number of subjects. This implies that no generalization of the findings can be applied to the asthmatic population. The small number of subject could explain the lack of significance between biological variables and quality of life. It is, however, important to highlight that the study was exploratory in nature and was intended as preliminary to larger studies with the attempt to select those biological markers who could show an association with impaired quality of life. In addition, we did not measure nasal NO; we feel confident, however, that ECP in the nasal wash provides similar information on the quality and magnitude of nasal inflammation. In addition, the discrepancy between normal lung function and lack of asthma control is only apparent; indeed, the control of asthma, specifically the ACT, which reflects the patient's perception of disease control, can be dissociated from the level of airway obstruction measured by the physician. 

The importance of measuring QoL in chronic allergic respiratory patients lies in the notion that the different pathophysiologic parameters that are commonly used as an index of severity are only part of a more general state of health. In addition to the common functional and clinical manifestations of asthma and rhinitis, these two conditions are typically associated with fatigue, disturbed sleep, and discomfort from therapies and medical equipment [4]. It is well known that asthma can cause a host of psychological problems such as anxiety, fear, addiction, or depression [15]. Rhinitis reduces academic and work performance, affects learning, and reduces the quantity and quality of sleep [16]. This stresses the need to assess the QoL of these patients by administration of specific instruments, for example, questionnaires. To this aim, we administered the Rhinasthma questionnaire, in which asthma and rhinitis are considered as different manifestations of the same disease [17]. 

Evaluation of NO in exhaled air has been proposed not only as a new experimental tool in translational medicine but also as a valuable clinical resource for asthma screening, diagnosis, and monitoring [18, 19]. Hence, its measurement could be used to assess asthma severity and the risk for exacerbations in asthmatic patients. In recent years, several studies have been performed to evaluate the relationship between eNO levels and parameters of lung function or other markers of airway inflammation [20]. In particular, it is well established that alveolar eNO levels are representative of peripheral bronchial inflammation [21–23]. As also inferred in several studies, the peripheral airways in asthmatics are the site of an inflammatory infiltrate that is not observed in nonasthmatic controls and, within asthmatic lungs, is more intense than in the central airways [24, 25].

In conclusion, the results of our explorative study provide additional insights into the factors that affect asthma control and the QoL associated with symptoms of upper and lower respiratory allergic diseases, suggesting a major role of abnormalities at the level of peripheral airways. Although novel, the findings of the current study should be interpreted with caution and confirmed by further studies specifically aiming at addressing the role of peripheral airways in impaired quality of life of uncontrolled asthmatics. We emphasize the need to perform the alveolar NO methodology to test whether in large population of uncontrolled asthmatics small airway abnormalities are the cause of impaired quality of life.

## Figures and Tables

**Table 1:** Lung function characteristics of the study subjects.

FEV_1, _% predicted	99 ± 21
FVC, % predicted	107 ± 19
FEV_1_/FVC, % predicted	98 ± 8
FEF_25_, % predicted	74 ± 33
FEF_50_, % predicted	87 ± 35
FEF_75_, % predicted	88 ± 26
FEF_25–75_, % predicted	80 ± 32

**Table 2:** Inflammatory markers from upper and lower airways.

FENO, ppb	21 ± 19
Alveolar NO, ppb	6.9 ± 5.9
pH of EBC	6.81 ± 0.1
ECP, ng/mL (nasal wash)	4.89 ± 3.4
Eosinophils, % (nasal wash)	13.5 ± 8.7
Neutrophils, % (nasal wash)	56 ± 21.9
Macrophages, % (nasal wash)	19.2 ± 19.2
Lymphocytes, % (nasal wash)	8.6 ± 5.1

EBC: exhaled breath condensate; ECP: eosinophil cationic protein.

## References

[1] National Institutes of Health NH Lung and Blood Institute: Global Strategy for asthma management and prevention (Updated 2011): Global Initiative for Asthma (GINA). http://www.ginasthma.org.

[2] Blaiss MS (2005). Rhinitis-asthma connection: epidemiologic and pathophysiologic basis. *Allergy and Asthma Proceedings*.

[3] Braido F, Baiardini I, Menoni S (2012). Patients with asthma and comorbid allergic rhinitis: is optimal quality of life achievable in real life?. *PLoS ONE*.

[4] Leynaert B, Neukirch C, Liard R, Bousquet J, Neukirch F (2000). Quality of life in allergic rhinitis and asthma: a population-based study of young adults. *American Journal of Respiratory and Critical Care Medicine*.

[5] Linneberg A, Henrik Nielsen N, Frølund L, Madsen F, Dirksen A, Jørgensen T (2002). The link between allergic rhinitis and allergic asthma: a prospective population-based study. The Copenhagen Allergy Study. *Allergy*.

[6] Braunstahl G-J, Fokkens W (2003). Nasal involvement in allergic asthma. *Allergy*.

[7] Bousquet J, Vignola AM, Demoly P (2003). Links between rhinitis and asthma. *Allergy*.

[8] Braido F, Baiardini I, Balestracci S (2009). Does asthma control correlate with quality of life related to upper and lower airways? A real life study. *Allergy*.

[9] Bousquet J, Khaltaev N, Cruz AA (2008). Allergic Rhinitis and its Impact on Asthma (ARIA) 2008 update (in collaboration with the World Health Organization, GA2LEN and AllerGen). *Allergy*.

[10] Nathan RA, Sorkness CA, Kosinski M (2004). Development of the Asthma Control Test: a survey for assessing asthma control. *Journal of Allergy and Clinical Immunology*.

[11] Pellegrino R, Viegi G, Brusasco V (2005). Interpretative strategies for lung function tests. *European Respiratory Journal*.

[12] American Thoracic Society, European Respiratory Society (2005). ATS/ERS recommendations for standardized procedures for the online and offline measurement of exhaled lower respiratory nitric oxide and nasal nitric oxide. *American Journal of Respiratory and Critical Care Medicine*.

[13] Horváth I, Hunt J, Barnes PJ (2005). On behalf of the ATS/ERS Task Force on Exhaled Breath Condensate. Exhaled breath condensate: methodological recommendations and unresolved questions. *European Respiratory Journal*.

[14] Scichilone N, Arrigo R, Paterno A (2011). The effect of intranasal corticosteroids on asthma control and quality of life in allergic rhinitis with mild asthma. *Journal of Asthma*.

[15] Urrutia I, Aguirre U, Pascual S (2012). Impact of anxiety and depression on disease control and quality of life in asthma patients. *Journal of Asthma*.

[16] Meltzer EO, Gross GN, Katial R, Storms WW (2012). Allergic rhinitis substantially impacts patient quality of life: findings from the Nasal Allergy Survey Assessing Limitations. *The Journal of Family Practice*.

[17] Baiardini I, Pasquali M, Giardini A (2003). Rhinasthma: a new specific QoL questionnaire for patients with rhinitis and asthma. *Allergy*.

[18] Alving K, Weitzberg E, Lundberg JM (1993). Increased amount of nitric oxide in exhaled air of asthmatics. *European Respiratory Journal*.

[19] Fischer A, Folkerts G, Geppetti P, Groneberg DA (2002). Mediators of asthma: nitric oxide. *Pulmonary Pharmacology and Therapeutics*.

[20] Jatakanon A, Lim S, Kharitonov SA, Chung KF, Barnes PJ (1998). Correlation between exhaled nitric oxide, sputum eosinophils, and methacholine responsiveness in patients with mild asthma. *Thorax*.

[21] van Veen IH, Sterk PJ, Schot R (2006). Alveolar nitric oxide versus measures of peripheral airway dysfunction in severe asthma. *European Respiratory Journal*.

[22] Williamson PA, Clearie K, Menzies D, Vaidyanathan S, Lipworth BJ (2011). Assessment of small-airways disease using alveolar nitric oxide and impulse oscillometry in asthma and COPD. *Lung*.

[23] Kelly HW (2010). Alveolar nitric oxide concentration, small airways inflammation, and targeted asthma therapy: are we there yet?. *Journal of Allergy and Clinical Immunology*.

[24] Johnson JR, Hamid Q (2012). Appraising the small airways in asthma. *Current Opinion in Pulmonary Medicine*.

[25] Tulic MK, Christodoulopoulos P, Hamid Q (2001). Small airway inflammation in asthma. *Respiratory Research*.

